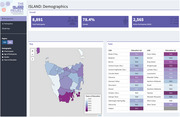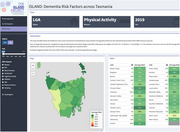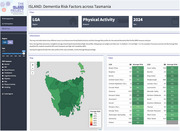# Using real world data to visualize longitudinal modifiable dementia risk factors in the community through an online interactive map

**DOI:** 10.1002/alz70860_104566

**Published:** 2025-12-23

**Authors:** Eddy Roccati, Alex Kitsos, Timothy Saunder, James C. Vickers

**Affiliations:** ^1^ Wicking Dementia Research and Education Centre, University of Tasmania, Hobart, TAS, Australia

## Abstract

**Background:**

Up to 45% of dementia cases could be prevented by addressing behavioural and lifestyle factors. Many of the modifiable risk factors offer distinct opportunities for targeted interventions, yet there is still a gap in how we address existing distributions of risk factor adherence in order to more effectively tailor interventions. Further, recognition of risk factor sociodemographic and geographic clusters can aid in effectively measuring the impact and efficacy of interventions. Using large‐scale epidemiological data, this study aimed to create a longitudinal, interactive and online dashboard for mapping modifiable dementia risk factors.

**Method:**

Six years of annual survey data were used (2019‐2024) from ISLAND (Island Study Linking Ageing and Neurodegenerative Disease); a public health initiative aiming to reduce dementia risk. Modifiable risk factors were assessed for each participant at each timepoint using the Dementia Risk Profile (DRP), a personalised traffic light tool based on latest evidence from the World Health Organisation. Weighted DRP averages were mapped using R, accessed as an interactive webpage by ISLAND's dedicated Community Advisory Group for initial feedback, then by the wider participant cohort (>13,500) and public. Participants (*n* = 8,891, 78.4% female) were drawn from a diverse range of Tasmanian local government areas and statistical areas.

**Result:**

There were observable geographic variations in education (range 13.4‐16.2 years, Figure 1), age (range 66.7‐72.4 years) and severable modifiable risk factors for dementia across Tasmania, Australia. Participants with higher ages tended to be in regional areas of the North‐West, whilst risk factor prevalence in Body Mass Index (Figure 2), Blood Pressure and Physical Activity were notably higher in the more remote West Coast region of Tasmania. Over time through the ISLAND initiative, all modifiable risk factors were seen to be shifting towards lower weighted averages on the DRP (Figure 3).

**Conclusion:**

Geographically visualizing modifiable risk factors in an online interactive format identified specific regions where targeted interventions are needed to reduce risk of dementia. Further, the efficacy of ISLAND to improve modifiable risk factors across Tasmania over time was able to be visually presented and accessible to the wide participant pool as well as the public.